# Are Abnormal Yolk Sac Characteristics Important Factors in Abortion Rates?

**Published:** 2012-06-19

**Authors:** Sanam Moradan, Mohammad Forouzeshfar

**Affiliations:** Department of Obstetrics and Gynecology, Amir University Hospital, Semnan University of Medical Sciences, Semnan, Iran

**Keywords:** Yolk Sac, Spontaneous Abortion, Abnormal

## Abstract

**Background:**

This study was undertaken to determine if there were different abortion rates between normal and abnormal yolk sacs.

**Materials and Methods:**

In this cohort study, the yolk sac characteristics of 193 consecutive pregnant women, of 5-6.5 weeks gestation, with normal body mass index (BMI) were prospectively evaluated. All patients underwent two-dimensional transvaginal ultrasonography, which was performed by the same sonographer. We considered the following yolk sac characteristics as normal for classification: diameter: 2-5 mm; round shape; absence of degenerative changes; equal number with embryos; echogenic rim and hypoechoic center. Yolk sacs that had diameters smaller than 2 mm or larger than 5 mm; a shape that was not round (i.e., oval or distorted); the presence of degenerative changes; hyper- or hypo-echogenic rim; hyperechoic center and unequal number with embryos were considered abnormal. Based on the above classification, patients were divided into two groups, study (abnormal yolk sac) and control (normal yolk sac). The study group contained 22 cases and the control group consisted of 164 cases. The primary outcome measure was the abortion rate between both groups. Chi-square and students’t test were used for data analysis.

**Results:**

A total of 193 cases were evaluated. We excluded 2 cases. Among the remaining 191 cases, 22 (11.51%) had abnormal yolk sacs of which spontaneous abortion occurred in 14 (63.63%) cases. In the control group, out of 169 (89.49%) cases, spontaneous abortion was noted in 6 (3.55%). There was a statistically significant difference in abortion rates between the two groups (p=0.000).

**Conclusion:**

According to the results of this study, it is obvious that abnormal yolk sac characteristics are associated with spontaneous abortion.

## Introduction

The first recognizable structure inside the gestational sac is the yolk sac, which should be detectable as a regularly rounded extra-amniotic structure when the gestational sac reaches dimensions of 8 to 10 mm. The normal biometric value of the yolk sac diameter during the first trimester should be an inner diameter of 3-6 mm.

Spontaneous abortion has been noted to occur in cases with no yolk sac, a yolk sac with dimensions over 6 mm or under 3 mm, an irregular shape (mainly wrinkled with indented walls), the presence of degenerative changes such as numerous calcifications that have decreased translucency of the yolk sac and the yolk sac number, which should be the same as the number of embryos as visualized by 2-D ultrasound (1).

In a normal pregnancy it should be possible to observe the yolk sac between 5-12 weeks of pregnancy or when it reaches 10 mm in size. Abnormal morphological appearance of the yolk sac and/or a size over 9 mm is suggestive of serious growth disorders of the fetus (2). Spontaneous abortion rates are significantly elevated in cases where the yolk sac volume falls outside the 5th to 95th percentile (3). Yolk sac diameters over 5.6 mm and the presence of an abnormal yolk sac shape visualized at initial sonography are associated with poor pregnancy outcome (4).

Pregnancies that have a mean yolk sac diameter equal or larger than 5 mm on early ultrasound are also associated with a threefold increased risk for first trimester loss, independent of maternal risk factors such as age, body mass index, polycystic ovary syndrome, smoking, and diabetes (5).

The lack of a yolk sac or a smaller than gestational age yolk sac diameter are indicative of pregnancies that may result in spontaneous abortion (6). Pregnancies with a very large yolk sac are generally always associated with poor outcomes (7).

This study was undertaken to determine if there were different abortion rates between normal and abnormal yolk sacs.

## Materials and Methods

This study was a cohort study carried out on 193 pregnant women who were between 5-6.5 weeks of gestation and with a body mass index (BMI) between 18-24. Patients were seen in Amir University Hospital, Semnan, Iran during May 2009 to May 2010. The method of sampling was convenient. Written informed consent was obtained from all patients. This study was approved by the Ethical Committee of Semnan University of Medical Sciences.

The following patients were excluded: those whose gestational age was more than 6.5 weeks, patients with diabetes, hypertension, or any systemic disease, those who expressed unwillingness to come for follow up visits, and known cases of Mϋllerian anomalies.

We performed two-dimensional transvaginal ultrasonography on 191 consecutive pregnant cases that were between 5-6.5 weeks of gestation, as part of the routine evaluation or other indication for ultrasonography. The size of the yolk sac (inner to inner diameter), shape, echogenicity of rim and center of sac, numbers of yolk sac and degenerative changes such as calcification were evaluated. For all cases, the two-dimensional transvaginal ultrasonography was performed by the same sonographer.

Yolk sacs that had the following characteristics were classified as normal: diameter between 2-5 mm, round shape, absence of degenerative changes, equal number with embryos, the presence of an echogenic rim and hypo-echoic center. Yolk sacs that had diameters smaller than 2 mm or larger than 5 mm, were not round (i.e., oval or distorted), had evidence of degenerative changes, hyper- or hypoechogenic rim, hyperechoic center, and unequal number with embryos were considered abnormal.

Based on the above mentioned criteria patients were divided into two groups, study (abnormal yolk sac) and control (normal yolk sac).

All cases were followed until delivery and the abortion rates (delivery before 20 weeks of gestation) were compared between the groups.

Data were expressed as percentage and compared using the chi-square and students’ t tests. Statistical analyses were performed with the Statistical Package for Social Sciences (SPSS, version 16.0).

## Results

The mean age of the study group was 28.1 ± 5.8 years and that of the control group was 28 ± 4.8 years, which was not statistically significant (p=0.876). During the study period a total of 193 cases were evaluated. There were two cases who did not return for their follow up visits, therefore we excluded them from the study. Out of 191 cases that were evaluated, 22 (11.51%) had abnormal yolk sacs of which spontaneous abortion occurred in 14 (63.63%). The control group consisted of 169 cases. Of these, spontaneous abortion occurred in 6 (3.55%). There was a statistically significant difference in abortion rates between the two groups (p=0.000).

**Fig 1:**
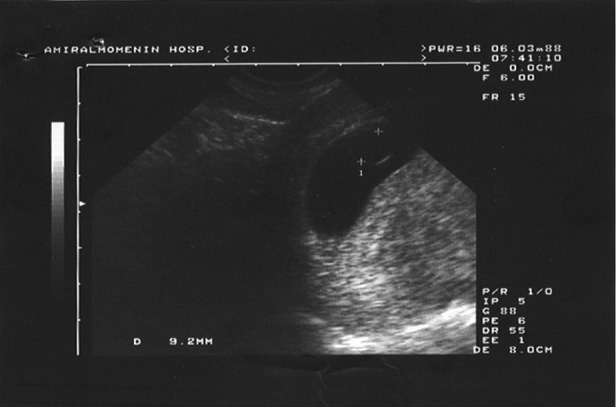
The largest yolk sac in a case with recurrent abortion.

With regards to abnormal yolk sac characteristics, 11 (50%) had yolk sacs that were greater than 5 mm; the largest was 9.2 mm in a patient who had four recurrent abortions (Fig 1). Spontaneous abortion occurred 10 (45.45%) who were between 6-8 weeks of pregnancy. The largest yolk sac with a normal, uneventful pregnancy was 6.6 mm in diameter (Table 1).

**Table 1:** Frequency of abortion rates of the study group based on yolk sac characteristics.


Characteristics	Number (%)	Abortion rates

**Size (>5 mm)**	11 (50)	10 (45.45)
**Distorted shape**	4(18.18)	2(9.9)
**Hypo-echoic rim**	4(18.18)	1(4.54)
**Two-yolk sac**	1(4.54)	0
**No yolk sac**	1(4.54)	1(4.54)
**Oval shape**	1 (4.54)	0


**Fig 2:**
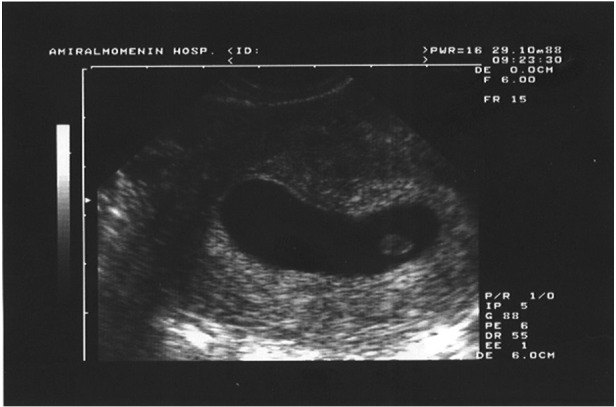
A distorted yolk sac (a case of abortion in this study).

Four (18.18%) had distorted yolk shape with spontaneous abortion in 2 (9.90%) who were between 6-8 weeks of pregnancy (Fig 2). However the other 2 (9.90%) continued their pregnancies to term with normal, live births (Table 1).

One (4.54%) case had two yolks, however her pregnancy was uneventful. In 1 (4.54%) case, no yolk sac was present and a spontaneous abortion occurred at nine weeks of pregnancy (Table 1).

One (4.54%) case had two yolks, however her pregnancy was uneventful. In 1 (4.54%) case, no yolk sac was present and a spontaneous abortion occurred at nine weeks of pregnancy (Table 1).

In 4 (18.18%) patients, the yolk sacs had hypo-echoic rims; all had long-term bleeding during the first trimester of pregnancy which was stopped by the use of progesterone preparations. With the exception of one abortion at seven weeks of pregnancy, the remaining pregnancies continued to term. In one case of this group, the pregnancy was terminated at 34 weeks because of oligohydramnios, but the fetus was normal and had no problems after delivery (Table 1).

In the control group, abortion occurred in 6 (3.55%) cases. Pregnancy continued to term in the remaining control cases (n=163; 96.44%). Preterm delivery occurred in 7 control patients; however all were more than 33 weeks pregnant. Of these, all neonates survived.

With regards to yolk sac characteristics of the control group, the sac diameters ranged from 2-4.9 mm, all of which had a round shape.

## Discussion

The yolk sac is the first structure of the gestational sac, which must be present when the mean gestational sac diameter is 13 mm or smaller in size (8). It is recommended in a patient at risk for poor pregnancy outcome to assess the yolk sac measurements prior to 12 weeks of gestation by transvaginal ultrasonography and repeat the assessment one to two weeks later when a discrepancy is detected in the first trimester (9, 10).

In this study we evaluated the characteristics of the first important structure of the gestational sac and its relation to the spontaneous abortion rate.

Pregnancies that have a mean yolk sac diameter equal or larger than 5 mm as visualized on early ultrasound are associated with a threefold increased risk of first trimester loss (5). Visualization of a large size yolk sac is a predictor of poor pregnancy outcome (11, 12).

In agreement with the above studies, the results of this study showed that yolk size was an important factor for prediction of spontaneous abortion. Yolk sacs larger than 5 mm at 5-6.5 weeks of pregnancy were a good indication that the probability of abortion was significantly high.

However, a very large yolk sac may exist in normal pregnancy and the presence of a yolk sac with a diameter of 8.1 mm in a viable pregnancy has been reported (13). In this study the largest yolk sac with a normal pregnancy outcome was 6.6 mm.

Larger yolk sac diameters may represent evidence of certain diseases and the pregnancy loss in these pregnancies is reflective of the presence of such underlying diseases. According to Ivanisević et al. in their study the researchers confirmed the presence of larger yolk sac diameters amongst type 1 diabetic women who were over six weeks of gestation (14).

Abnormal yolk sac shape are associated with abnormal pregnancy outcome with a sensitivity of 29%, specificity of 95%, positive predictive value of 47% and negative predictive value 90.5% (4, 15). Identification of abnormal yolk sacs may suggest the death of one or all embryos (16). The current study showed that among 4 (18.18%) cases with distorted yolk sacs spontaneous abortion happened in 2 (9.90%). However the remaining 2 (9.90%) cases with oval-shaped yolk sacs had normal pregnancies.

Failure to detect the presence of a yolk sac before the detection of an embryo in the first trimester by ultrasound is suggestive of an abnormal intrauterine pregnancy (17). In this study there was one (4.54%) case that had no yolk sac in the eighth week of pregnancy. This case aborted the fetus after one week.

Usually there is one yolk sac in a single pregnancy and the number of sacs must equal the number of embryos. During the first trimester of a dichorionic twin pregnancy the yolk sacs are always separated by a septum. They are not separated in a monochorionic twin pregnancy (11). In this study there was one (4.54%) case which had one embryo and two yolk sacs, whose pregnancy had a normal outcome.

Usually a normal yolk sac has an echogenic rim and hypoechoic center. In the current study there were 4 (18.18%) with hypoechoic rims, all experienced long term bleeding in the first trimester, however only one case had an abortion. It was probable that this particular characteristic of the yolk sac was not an important factor for an abortion.

## Conclusion

We have concluded that abnormal yolk sac characteristics are associated with spontaneous abortion. In this study abortion occurred in 90.9% of cases where the yolk sac was of an abnormal size and in 50% of those with distorted shape yolk sacs.

Thus, it is presumed that among the yolk sac characteristics, a large-size yolk and distorted shape are the most important factors for early pregnancy loss. Evaluation of the importance of other criteria needs additional studies with larger numbers of cases.
